# A pilot feasibility and acceptability study of an Internet-delivered psychosocial intervention to reduce postoperative pain in adolescents undergoing spinal fusion

**DOI:** 10.1080/24740527.2021.2009334

**Published:** 2022-04-13

**Authors:** Caitlin B. Murray, Anthea Bartlett, Alagumeena Meyyappan, Tonya M. Palermo, Rachel Aaron, Jennifer Rabbitts

**Affiliations:** a Department of Anesthesiology & Pain Medicine, University of Washington, Seattle, Washington, USA; b Center for Child Health, Behavior and Development, Seattle Children’s Hospital, Seattle, Washington, USA; c School of Social and Political Science, The University of Edinburgh, Edinburgh, UK; d School of Medicine, Virginia Commonwealth University, Richmond, Virginia, USA; e Department of Physical Medicine and Rehabilitation, Johns Hopkins University School of Medicine, Baltimore, Maryland, USA; f Seattle Children’s Hospital, Center for Clinical and Translational Research, Seattle, Washington, USA

**Keywords:** adolescent scoliosis, acute pain, chronic pain, psychosocial intervention

## Abstract

**Background:**

Spinal fusion surgery is a common and painful musculoskeletal surgery performed in the adolescent population. Despite the known risk for developing chronic postsurgical pain, few perioperative psychosocial interventions have been evaluated in this population, and none have been delivered remotely (via the Internet) to improve accessibility.

**Aims:**

The aim of this single-arm pilot study was to evaluate the feasibility and acceptability of the first Internet-based psychological intervention delivered during the perioperative period to adolescents undergoing major spinal fusion surgery and their parents.

**Methods:**

Thirteen adolescents (M age = 14.3; 69.2% female) scheduled for spine fusion surgery and their parents were provided access to the online psychosocial intervention program. The program included six lessons delivering cognitive-behavioral therapy skills targeting anxiety, sleep, and acute pain management during the month prior to and the month following surgery. Feasibility indicators included recruitment rate, intervention engagement, and measure completion. Acceptability was assessed via quantitative ratings and qualitative interviews.

**Results:**

Our recruitment rate was 81.2% of families approached for screening. Among participating adolescent–parent dyads, high levels of engagement were demonstrated (100% completed all six lessons). All participants completed outcome measures. High treatment acceptability was demonstrated via survey ratings and qualitative feedback, with families highlighting numerous strengths of the program as well as areas for improvement.

**Conclusions:**

These findings suggest that this online psychosocial intervention delivered during the perioperative period is feasible and acceptable to adolescents and their parents. Given favorable feasibility outcomes, an important next step is to evaluate the intervention in a full-scale randomized controlled trial.

## Abstract (fr)

**Contexte:**

La chirurgie de fusion vertébrale est une chirurgie musculo-squelettique courante et douloureuse pratiquée chez la population adolescente. Malgré le risque connu de développer une douleur post-chirurgicale chronique, peu d’interventions psychosociales périopératoires ont été évaluées chez cette population, et aucune n’a été dispensée à distance (par Internet) pour améliorer son accessibilité.

**Objectifs:**

L’objectif de cette étude pilote à un seul volet était d’évaluer la faisabilité et l’acceptabilité de la premiére intervention psychologique sur Internet destinée aux adolescents subissant une chirurgie majeure de fusion vertébrale et à leurs parents, dispensée pendant la période périopératoire.

**Méthodes:**

Treize adolescents (âge M = 14,3 ; 69,2 % de filles) devant subir une chirurgie de fusion vertébrale et leurs parents ont eu accés au programme d’intervention psychosociale en ligne. Le programme comprenait six leçons permettant d’acquérir des compétences de thérapie cognitivo-comportementale ciblant l’anxiété, le sommeil et la prise en charge de la douleur aiguë pendant le mois précédant et le mois suivant la chirurgie. Les indicateurs de faisabilité comprenaient le taux de recrutement, l’engagement dans l’intervention et la réponse aux questionnaires de mesure des résultats. L’acceptabilité a été évaluée au moyen d’évaluations quantitatives et d’entretiens qualitatifs.

**Résultats:**

Notre taux de recrutement était de 81,2 % des familles approchées pour le dépistage. Parmi les dyades adolescents-parents participantes, des niveaux élevés d’engagement ont été démontrés (100 % ont terminé les six leçons). Tous les participants ont rempli les questionnaires de mesure des résultats. Une acceptabilité élevée du traitement a été démontrée par le biais de sondages et de rétroaction qualitative, les familles mettant en évidence de nombreux points forts du programme ainsi que les points à améliorer.

**Conclusions:**

Ces résultats indiquent que cette intervention psychosociale en ligne dispensée pendant la période périopératoire est faisable et acceptable pour les adolescents et leurs parents. Étant donné les résultats de faisabilité favorables, une prochaine étape importante consistera à évaluer l’intervention dans le cadre d’un essai contrôlé randomisé à grande échelle.

## Introduction

Spinal fusion surgery is a common and painful musculoskeletal surgery performed in the adolescent population for idiopathic spinal deformities (e.g., scoliosis). Studies demonstrate that most youth undergoing spinal fusion experience moderate to high pain intensity immediately after surgery and are at risk for having persistent postsurgical pain.^1–6^ Of particular concern is that up to 20% of youth have persistent postsurgical pain, often accompanied by functional limitations and impairments in health-related quality of life.^7–10^ Identifying risk factors for persistent postsurgical pain is an active area of investigation, and studies have found that acute pain in the immediate postsurgical period as well as psychosocial risk factors predict the transition from acute to chronic postsurgical pain.^7,8,11–14^

Building from this work, Rabbitts et al.^15^ proposed a biopsychosocial conceptual model of the transition from acute to chronic postsurgical pain (CPSP) in adolescents, defined as pain that impacts quality of life persisting at least 3 months after surgery^11,16^ They identified several modifiable psychosocial risk factors that can be targeted perioperatively to reduce the occurrence of CPSP and improve health outcomes, including adolescent anxiety, sleep disruption, parental distress, and low pain self-efficacy. Psychosocial interventions are urgently needed to improve postsurgical pain outcomes for youth and to prevent the transition from acute to chronic postsurgical pain. To date, a small body of literature exists on psychological interventions for postoperative pain in youth. In a recent systematic review by Davidson et al.^17^ psychological interventions as a whole were effective in reducing children’s self-reported pain in the short term. However, unfortunately, data on the effects of psychological interventions on longer-term pain outcomes (including CPSP) were limited.^17^

Opportunities exist before and following surgery to provide psychological interventions to youth and families, yet resources are not typically available in the perioperative model to provide this type of care.^18^ Qualitative research with adolescents, families, and health care providers identified a need for and gaps around psychosocial preparation and pain self-management for youth undergoing major surgery.^19^ However, families identified limited time and the burden of perioperative appointments as potential barriers to participating in a perioperative program, endorsing interest in web-based or mobile applications to enable participation.^19^ Digital health interventions using web-based and mobile applications have been successfully used to deliver psychological interventions to other pediatric populations,^20,21^ and thus we anticipated they would also be relevant and feasible for youth undergoing spinal fusion surgery.

The primary aim of this single-arm pilot study was therefore to evaluate the feasibility and acceptability of an Internet-based psychological intervention delivered during the perioperative period to youth undergoing spinal fusion for idiopathic spinal deformities and their parents. Intervention strategies followed a cognitive-behavioral framework and addressed three primary targets: anxiety/distress (adolescent and parent), sleep, and acute pain management, with specific skills training delivered during the pre- and postoperative periods. We hypothesized that intervention feasibility would be demonstrated through (1) reaching at least a 50% recruitment rate of the study population, (2) high treatment engagement as shown by completion of at least five of six lessons and telephone calls with study coaches by 75% of the sample, and (3) retaining at least 80% of youth in the study with complete assessments. We also expected that participants would rate the intervention as highly acceptable on self-report measures and qualitative interviews but may also have suggestions for modifying the program.

## Materials and Methods

### Participants and Setting

Participants included 13 adolescents (69.2% female) ages 12 to 17 years who were scheduled for elective inpatient spinal surgery (spine fusion) at a university-affiliated children’s hospital in the northwestern region of the United States and their parent/caregiver (i.e., 13 adolescent–parent dyads, *n* = 26 participants). The standard of care at this hospital included a preoperative visit at the anesthesia clinic to complete preanesthesia medical evaluation; a preoperative appointment at the surgery clinic to complete medical history, physical exam, and surgical consent, a postprocedure phone call on postdischarge day 3; and surgery appointments at approximately 1, 3, 6, and 12 months postsurgery.

Participants were recruited from July 2017 to November 2017. Data collection for the study was completed in April 2018. The study was approved by the Seattle Children’s Hospital Institutional Review Board, Seattle Washington (IRB approval Number STUDY00000679). Parents provided written consent and adolescents provided written assent prior to any research procedures.

### Recruitment

Study staff identified adolescents with scheduled spine fusion surgery meeting inclusion criteria from automated reports generated from the electronic medical record at a university-affiliated children’s hospital. Potential youth participants and their parents were mailed a study flyer and invitation letter informing them of study eligibility. Study staff contacted families at least 4 to 8 weeks before surgery to complete eligibility screening and enrollment via phone.

### Inclusion/Exclusion Criteria

Inclusion criteria were (1) ages 10 to 18 years and (2) scheduled for elective spine fusion surgery for idiopathic scoliosis or kyphosis. Potential participants were excluded if they (1) had chronic or complex health conditions such as cancer, neurodegenerative or neurological disorders, or a prior history of major surgery; (2) had major psychiatric condition requiring inpatient care; (3) had a cognitive or developmental delay; (4) were unable to read English well enough to complete questionnaires or the study intervention; or (5) did not have personal Internet access on any device (e.g., phone, computer).

### Trial Design and Procedures

This was a single-arm pilot feasibility study of an Internet-based psychosocial intervention including presurgery and postsurgery intervention phases. The preoperative intervention phase began 4 to 6 weeks before scheduled surgery and the postoperative phase began 1 week postsurgery. Youth and parents completed a baseline assessment before receiving the intervention (T1: baseline/4–8 weeks before surgery). Participants completed a second and third assessment following completion of the presurgical and postsurgical intervention phases, respectively (T2: midintervention/1 week before surgery; T3: postintervention/6–8 weeks postsurgery). A final follow-up assessment was completed 3 months postsurgery (T4).

All study assessments were completed online using REDCap (Research Electronic Data Capture)^22^ via e-mail/text survey links and included standardized measures. REDCap is a secure, web-based application designed to support data capture for research studies. Participants received e-mail or phone reminders by study staff to complete survey measures. Participants received gift card incentives for completing study assessments.

#### Description of Intervention

All adolescents and parents were provided access to the Internet-based psychosocial intervention program. There were separate adolescent and parent versions of the program. The intervention was built in REDCap, with links to lessons sent every 1 to 2 weeks to participants via e-mail in each intervention phase at the beginning of the lesson window (see Table 1). The core components of the program followed a cognitive-behavioral therapy framework where participants learn about the link between behavior, thoughts, and feelings and were intended to address three primary targets: anxiety (teen and parent), sleep, and pain coping skills. In the preoperative period, the core strategies included thought restructuring, thought stopping, relaxation strategies (e.g., deep breathing, imagery), and sleep hygiene, and in the postoperative period the core strategies included behavioral activation, activity pacing, and several relaxation strategies (e.g., mindful breathing, “mini relaxation”). Postoperative content also included select cognitive-behavioral strategies taught in the preoperative intervention phase that were reviewed and applied to the postoperative context (e.g., thought replacement, sleep hygiene; see Table 1 for further details).

**Table 1.** Summary of adolescent and parent intervention timing and content

Lesson	Delivery/timing	Teen content/skills	Parent content/skills
**Preoperative intervention phase**			
Lesson 1: Preparing for Surgery	4–6 weeks prior to surgery	Information on preparing for surgery Psychoeducation on relaxation strategies and training in deep breathing Information about the role of sleep in recovery and provision of strategies to improve sleep habits	Information on preparing for surgery Psychoeducation on relaxation strategies and training in deep breathing Information about the role of sleep in recovery and provision of strategies to improve teen sleep habits
Lesson 2: Coping with Stress before Surgery	2–4 weeks prior to surgery	Psychoeducation on cognitive strategies Training in cognitive skills (thought replacement, thought stopping) and “54321 mindfulness” (relaxation skill using five senses for grounding)	Psychoeducation on cognitive strategies Training in cognitive skills (thought replacement, thought stopping) and “54321 mindfulness” (relaxation skill using five senses for grounding) Strategies to enhance parent–child communication
Lesson 3: Getting Ready for the Hospital	1–2 weeks prior to surgery	Information on preparing for the hospital including developing a social connection plan Training in additional relaxation (i.e., imagery) and cognitive (i.e., distraction) strategies for managing anxiety and pain	Information on preparing for the hospital including developing a social connection plan Training in additional relaxation (i.e., imagery) and cognitive (i.e., distraction) strategies for managing anxiety and pain Information on preparing for hospital discharge
**Postoperative intervention phase**			
Lesson 4: Coping at Home after Surgery	1 week postsurgery	Introduction to behavioral activation and training in pleasant activity scheduling to improve mood Review of thought restructuring Training in mindful breathing	Introduction to basic principles of self-care Introduction to behavioral activation and training in pleasant activity scheduling to improve teen mood Review of restructuring thoughts Training in mindful breathing
Lesson 5: Return to Activities and School	2–4 weeks postsurgery	Review of strategies to improve sleep habits Information about preparing to make transition back to school Goal setting and training in strategies to gradually increase physical activities (i.e., activity pacing) Training in “mini relaxation” and using relaxation strategies in school	Review of strategies to improve sleep habits Information about preparing to teen to make transition back to school Goal setting and training in strategies to gradually increase teen’s physical activities (i.e., activity pacing) Operant strategies to facilitate teen’s return to activities (praise, reinforcement) Training in “mini relaxation”
Lesson 6: Long-Term Recovery	6–8 weeks postsurgery	Review of skills Relapse prevention	Review of skills Relapse prevention

The content was developed by an interdisciplinary team including patient representatives as well as experts in pediatric perioperative and pain medicine, pediatric psychology, and remotely delivered psychological interventions. The content was further informed by patient, parent, and provider stakeholder input.^19^ The program included six core lessons developed separately for children and parents: three delivered during the presurgical phase and three delivered during the postsurgical phase. An accompanying behavioral assignment was given at the end of each lesson to assist participants in skills practice and acquisition (e.g., “Practice your deep breathing skill several times each day”); assignment completion was not tracked. Content included text and pictures and provided a combination of didactic instruction and narrative examples incorporated throughout the program. The focus of each lesson was relevant to the timing of the perioperative period (e.g., “Getting ready for the hospital” and “Coping at home after surgery”).

To supplement the Internet intervention, support was provided through coaching calls (5–10 min by phone) following each lesson. Study coaches were PhD-level postdoctoral psychology fellows with previous experience in cognitive-behavioral therapy for pain management. Coaches encouraged participants to practice the skills taught within the course and identify ways to apply skills to their individual/family context. To standardize interactions with participants, coaches followed a study coach manual and were supervised by a licensed clinical psychologist.

Lessons were designed to take approximately 20 to 30 min to complete for a total treatment time of approximately 120 to 180 min with an additional 30 to 60 min of coach contact time.

##### Adolescent Version

The adolescent version of the psychosocial intervention provided instruction in cognitive-behavioral strategies intended to ameliorate anxiety/distress, improve sleep, and reduce pain. Specifically, adolescent lessons included psychoeducation on pain and recovery and instruction in cognitive skills (e.g., recognizing stress, thought restructuring), relaxation training (e.g., deep breathing, mindfulness), behavioral strategies (e.g., activity pacing, pleasant activity scheduling), and sleep hygiene (optimizing sleep duration and sleep quality).

##### Parent Version

The parent version of the intervention provided instruction in cognitive-behavioral strategies to reduce parent anxiety/distress and support their teen’s recovery and use of coping strategies. Specifically, parent lessons delivered instruction in cognitive strategies (e.g., thought restructuring), relaxation strategies (e.g., deep breathing), as well as on parent preparation before surgery (e.g., gathering information, talking to your teen before surgery), strategies for recovery following surgery (e.g., the importance of parent self-care), and operant strategies to support their teen’s use of coping skills (e.g., reinforcement, praise).

### Measures

#### Demographic Characteristics

Parents reported on their relationship to the adolescent, household income, education, and race/ethnicity. Parents also reported on their child’s sex, age, and race.

#### Program Feasibility

Program feasibility was assessed using (1) study recruitment/enrollment statistics, (2) intervention engagement, and (3) rate of completion of study time points and assessments. The recruitment rate and enrollment rate were computed from schedule and screening data. Treatment engagement was measured by the number of completed intervention lessons in the presurgical and postsurgical program and the number of completed coaching calls. Retention and assessment completion were measured by the percentage of youth completing each of the four assessment time points and the completion of survey measures.

#### Program Acceptability

Program acceptability was assessed using quantitative and qualitative data. Youth and parent participants completed a five-item program evaluation survey to provide quantitative ratings of program acceptability, including items related to convenience, usefulness, accessibility, and understandability. Participants completed the program evaluation survey following completion of the presurgical and postsurgical lessons (i.e., as part of their T2 and T3 assessments). All items were scored on a 5-point Likert rating scale, with higher scores indicating greater acceptability and satisfaction.

All parent and teen participants who completed the online program were invited to participate in an optional qualitative interview to assess program satisfaction and obtain feedback. Of the 12 dyads (one family was withdrawn; see ”Intervention Engagement and Adherence” below for further details), one family could not be contacted and four families declined participation in the qualitative interviews. Thus, a total of seven parents and seven adolescents agreed to participate and completed interviews. Qualitative interviews included a semistructured set of questions and probes intended to elicit participants’ experiences with and feedback on the program’s components (e.g., skills) and general structure (e.g., coaching calls) of the program. Interviews were conducted by a postdoctoral fellow in pediatric psychology. Parents and adolescents were interviewed separately by phone and all interviews were audio-recorded and transcribed.

#### Questionnaire Measures

Primary measures included adolescent report on pain severity, pain interference, and health-related quality of life. Secondary measures included adolescent report on anxiety (State–Trait Anxiety Inventory–State Scale; 20 items^23^) sleep quality (Adolescent Sleep Wake Scale; 10-item short version^24^), and pain coping skills (Pain Coping Questionnaire; 39 items^25^). Parents also reported on parental distress using the Brief Symptom Inventory (18-item version^26^). Because the current study aimed to determine feasibility, we examined the rate of measure completion at each time point and present baseline data on the following primary measures to describe the study sample.

##### Pain Intensity and Interference

Youth completed one item from the PROMIS Pain Intensity scale (Pediatric Version)^27^ measuring average pain intensity over the previous 7 days with an 11-point numerical rating scale ranging from 0 to 10.^28^ To capture pain interference, youth completed the PROMIS Pain Interference scale (PROMIS-PI; Pediatric version),^27^ an eight-item measure assessing the degree to which pain interfered with youths’ daily activities in the past 7 days.

##### Health-Related Quality of Life

Health-related quality of life was assessed with the widely used Pediatric Quality of Life Inventory (PedsQL) Short Form measuring self-reported physical and psychosocial health in the preceding 7 days (Acute Version^29^). Youth indicate perceived difficulty in physical, school, social, and emotional health domains with responses indicated on a 5-point scale, ranging from *never* to *almost always*. The measure yields a total health score ranging from 1 to 100 (higher scores indicate better health-related quality of life).

### Data Analysis Plan

Data analyses were conducted using IBM SPSS Statistics for Windows, Version 27.0 (Armonk, NY: IBM Corp). For our primary aim, we conducted descriptive statistics to examine indicators of program feasibility and participants’ quantitative ratings of program acceptability. Specifically, we computed the percentage of the available sample recruited and enrolled in the study as well as percentage completion of the four assessment time points and completion of survey measures. Treatment engagement was measured by the number of completed intervention lessons (out of six) as determined by REDCap usage data. We used our tracking database to compute the number of completed coaching calls for each adolescent and parent participant and calculated the proportion of the sample completing at least five calls.

Qualitative interviews were coded using semantic thematic analysis following the guidelines of Braun and Clarke.^30^ The qualitative coding team was composed of a psychologist with experience in cognitive-behavioral therapy and pediatric pain management, a pain medicine physician with experience in working with youth undergoing major surgery including spine fusion, and an undergraduate student in psychology. Before initiating coding, the team reviewed the interview transcripts to become familiar with the data. Two primary coders created initial codes by organizing text into meaningful groups using NVivo v.10.^31^ Next, the two coders worked together to group similar codes into subcategories and then the subcategories were grouped into overarching themes. At each stage of coding, the codes, categories, and themes were recorded in a codebook that included operational definitions and representative quotes. Using this codebook, the coders worked together to achieve consensus. When there was disagreement, a third study team member arbitrated. Using this process, 100% agreement was achieved at each stage of coding.

#### Sample Size

Because this was a pilot study, a sample size calculation was not performed. Consulting guidelines for pilot studies^32,33^ a sample size of 13 adolescent–parent dyads (*n* = 26) was deemed adequate to provide information on feasibility and acceptability.

## Results

### Participant Characteristics

Baseline characteristics of the sample are summarized in Table 2. Participants included 13 adolescent–parent dyads. Adolescents were between the ages of 12 and 17 years (*M* = 14.3, SD = 1.4) and predominantly female (69.2%) and white (69.2%). At baseline (prior to the surgery or intervention), adolescents reported mild pain intensity (*M* = 3.00, SD = 0.41). PROMIS-derived *T*-scores indicated adolescents reported slightly elevated pain interference (*M* = 54.3, SD = 7.8), with around one-fourth (23.1%) reporting pain interference levels 1 SD above the mean (i.e., a *T*-score of ≥60). Moreover, 38.1% (*n* = 5) reported significant impairments in health-related quality of life (i.e., total score on PedsQL <74.9).

**Table 2.** Sample baseline characteristics (*n* = 13 parent–adolescent dyads)

**Adolescent characteristics**	
Age (years), *M* (SD)	14.3 (1.4)
Range	12−17
Sex (female), *n* (%)	9 (69.2)
Race/ethnicity, *n* (%)	
Hispanic/Latinx	1 (7.7)
Black	1 (7.7)
Asian	1 (7.7)
American Indian or Alaska Native	1 (7.7)
White	9 (69.2)
VAS average pain intensity, *M* (SD)	3.0 (0.4)
PROMIS pain interference, *M* (SD)*	54.3 (7.8)
PedsQL total, M (SD)	74.1 (18.7)
*n*, % cutoff <75	5 (38.1)
**Parent characteristics**	
Parent/caregiver relation to adolescent, n (%)	
Mother	10 (76.9)
Father	2 (15.4)
Grandparent	1 (7.7)
Race/ethnicity, *n* (%)	
Hispanic/Latinx	0 (0.0)
Black	0 (0.0)
Asian	1 (7.7)
American Indian or Alaska Native	1 (7.7)
White	11 (84.6)
Marital status, *n* (%)	
Married or remarried	9 (69.2)
Divorced	1 (7.7)
Widowed	1 (7.7)
Never married	2 (15.4)
Highest level of education completed, *n* (%)	
High school or less	4 (30.8)
Vocational or trade school	4 (30.8)
College or university	3 (23.1)
Graduate degree/professional school	2 (15.4)
Annual household income, *n* (%)	
≤$29,999	1 (7.7)
$30,000–$49,999	0 (0.0)
$50,000–$69,999	1 (7.7)
$70,000–$100,999	5 (38.5)
More than $100,999	6 (46.2)

*VAS, Visual Analogue Scale, 0–10; PROMIS, Patient-Reported Outcomes Measurement Information Systems, scores based on a population mean of 50 with a SD of 10; PedsQL, Pediatric Quality of Life Inventory (based on the established cut point of 1 standard deviation below the population mean, values below 74.9 indicate impairment in health-related quality of life).

### Feasibility

#### Recruitment and Enrollment Rate

shows a CONSORT (Consolidated Standards Of Reporting Trials) diagram depicting the flow of study participants and summarizing recruitment and engagement at each step of this pilot feasibility trial. Study staff identified 19 potentially eligible families via surgery schedules and electronic medical records of adolescents. The research staff was unable to reach 3 of the potentially eligible families. Of the 16 potential families who were reached, all met inclusion criteria and were invited to participate. Three families declined participation, with reasons including lack of interest (*n* = 2) and lack of time (*n* = 1). The remaining 13 families or parent–adolescent dyads (*n* = 26) enrolled in the study (overall recruitment/enrollment rate = 68.4%; enrollment rate among those approached = 81.2%). Thus, we exceeded our first indicator of feasibility based on our recruitment rate metric of at least 50%.

#### Intervention Engagement and Adherence

Engagement in the online intervention program was excellent, with all 13 adolescent–parent dyads (100%) completing all three lessons of both the adolescent and parent preoperative intervention phase. One family was withdrawn from the study before completing the second postoperative phase of the intervention due to the adolescent needing to undergo an unanticipated second surgery (reoperation of the spine). Of the remaining 12 dyads, 100% completed all three lessons of the postoperative intervention phase. During the intervention, 91.7% of adolescents (*n* = 11/12) and 83.3% of parents (*n* = 10/12) completed at least five out of six telephone coaching calls (adolescents: *M* = 5.3, SD = 1.4, range = 1–6; parents: *M* = 5.2, SD = 1.5, range = 1–6). Thus, we met our second metric for feasibility based on our treatment engagement exceeding a 75% completion rate for lessons and coaching calls.

#### Assessment Completion

**Figure 1.**
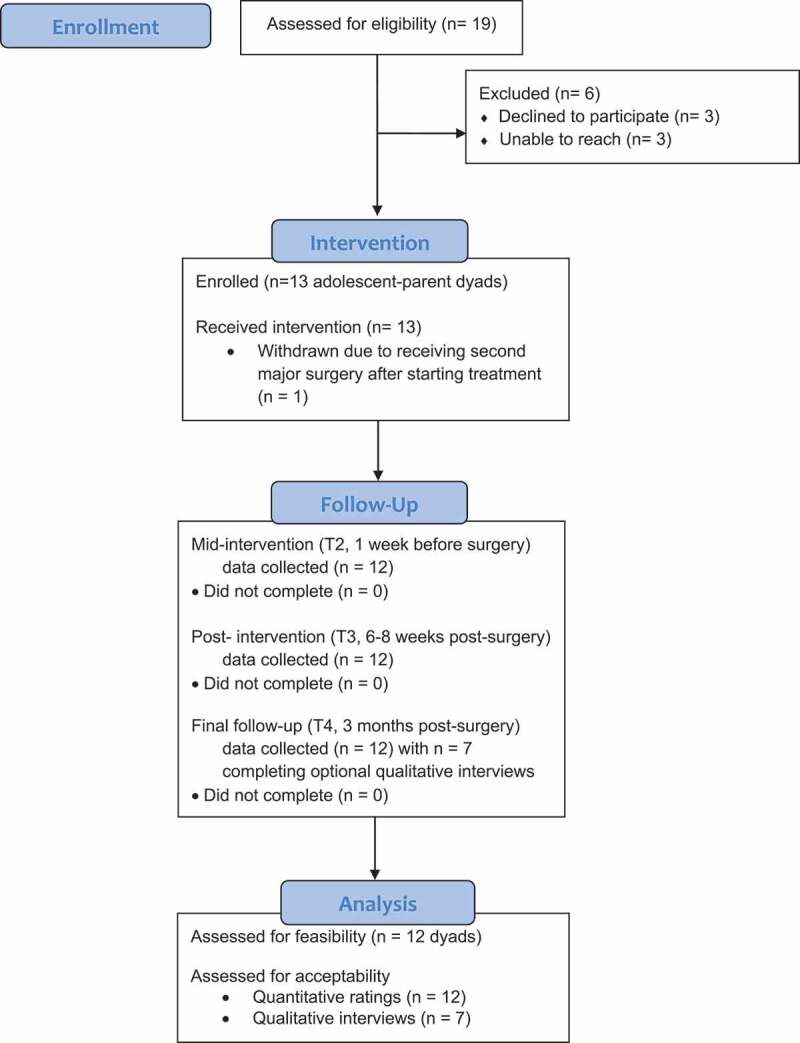
Consort diagram depicting flow through the study from recruitment to analysis.

Assessment completion was excellent (see Figure 1 CONSORT diagram). All 13 (100%) enrolled dyads completed their baseline (T1, 4–8 weeks before surgery) and second assessments (T2, 1 week before surgery). As described above, one family was withdrawn from the study prior to the postoperative intervention phase. Of the remaining 12 dyads, 100% completed the third (T3, postintervention/6–8 weeks postsurgery) and fourth (T4, 3-month follow-up/postsurgery) follow-up assessments. Thus, we exceeded our third feasibility metric of at least 80% for retention of the sample and assessment completion.

### Intervention Acceptability

#### Quantitative Feedback

Overall, adolescents and parents rated the intervention to be acceptable. The mean item-level scores on the program evaluation survey ranged from 3.2 to 4.9 for the presurgical program and from 3.0 to 4.6 for the postsurgical program, corresponding to moderate to high ratings of acceptability on average (see Table 3; item range 0−5).

**Table 3.** Program evaluation: Quantitative ratings on intervention acceptability

Questions	Parent presurgery program	Teen presurgery program	Parent postsurgery program	Teen postsurgery program
1. Did the program come at a good or convenient time? (0 =* very inconvenient* to 5 =* very convenient*)	4.0 (0.9)	3.8 (1.1)	4.0 (1.0)	3.3 (1.1)
2. How easy or difficult was it to understand the information presented? (0 =* very difficult* to 5 =* very easy*)	4.8 (0.4)	4.5 (0.8)	4.6 (0.5)	4.2 (0.8)
3. Did you like accessing the information online? (0 =* did not like it at all* to 5 =* liked it a lot*)	4.9 (0.3)	4.0 (1.2)	4.6 (0.9)	3.8. (1.3)
4. How useful or relevant was the program content overall? (0 =* not at all useful* to 5 =* very useful*)	4.2 (1.1)	3.6 (0.9)	4.3 (0.7)	3.3 (1.1)
5. If you did at least one, how useful were the telephone coaching calls? (0 =* not at all useful* to 5 =* very useful*)	4.2 (1.1)	3.2 (1.6)	3.5 (1.2)	3.0 (1.0)

#### Qualitative Feedback

As shown in Table 4, ten themes emerged that describe participants’ experiences using the psychosocial intervention. Identified themes were organized into the following topic areas for reporting purposes: (1) intervention components (i.e., lesson/skills), (2) general program structure, and (3) suggestions for improvement.

**Table 4.** Program evaluation: Qualitative feedback on intervention acceptability

Topic	Themes	Representative participant quotes
Intervention components (skills/lessons)	Cognitive and relaxation strategies helped adolescents and parents cope with stress	“I had thoughts like, ‘Oh my gosh, this is going to be so overwhelming,’ or, ‘This is going to take forever,’ stuff like that, and I definitely reframed those to, ‘Well, they said it’s going to be better every week, so I’m going to rely on that.’” (ID9, parent) “Sometimes with the picturing you’re in a different place … those [strategies] were like the main things that helped me through surgery.” (ID9, teen)
	Cognitive and relaxation strategies helped adolescents cope with pain during recovery	“I used some distraction techniques when she was in a lot of pain … encouraging her to talk about school or something else outside of the four walls of the hospital room.” (ID1, parent) “Deep breathing, like, calms me down and usually makes my pain feel a whole lot better.” (ID11, teen)
	Strategies for improving sleep were beneficial before surgery and during recovery	“I would usually go to bed at the same time and I would play music usually to go to sleep … and I still kind of do that to just relax.” (ID6, teen) “He got more sleep than he would have in the weeks going up to the surgery because of the lessons … like, I was really worried about how we were going to keep him on a schedule over all those weeks home because the surgery … but he just slept at the normal times and kept a schedule.” (ID9, parent)
	Activity pacing and goal setting used to gradually return to regular activities	“We would do little goals here and there, like one day let’s walk to the mailbox … and then let’s walk around the kitchen. … And then another [goal] was try to walk in the backyard.” (ID6 parent) “After surgery, gradually getting back into the things I did was helpful … instead of just rushing right back into it. For me we started with half days at school, so I’d go to part of my classes and go home instead of trying to do all 6 periods of my classes.” (ID3, child)
	Parents valued strategies to encourage self-care	“Self-care—having that was a good reminder … because you you’re so focused on taking care of your child that you kind of put yourself to the side … having that was a good reminder just to kind of give yourself a chance to recharge a little bit.” (ID3, parent) “Self-care was helpful, especially when it talked about managing your expectations for your recovery and giving yourself some grace.” (ID1, parent)
Program structure	Families found narratives relatable and validating	“It was like we were reading a diary of what we were going through. … ” (ID6, parent) “It helped me to know that there’s other people that got the surgery because it kind of feels like it’s just me … like, everyone around me doesn’t even know what scoliosis is.” (ID9, teen)
	Families appreciated flexibility of online program	“[We liked] the flexibility because the weeks after surgery … we just wouldn’t have been able to participate if it had to be during business hours.” (ID9, parent) “Sometimes I would get really tired, and I could pause it and go back to it later … so I liked doing it when I had nothing else to do.” (ID6, teen)
Suggestions/improvements	Rethink timing and reduce length of first postsurgical lesson	I felt that you guys needed to maybe wait a little longer [to send the first postsurgical lesson] … it was literally like the next day when we were home, and I’m like, “I’ve already got my hands full!” (ID2, parent) “Sometimes [the timing of lessons] was good and sometimes it wasn’t. Like right after the surgery maybe not as long of lessons … it was really hard to do. I was hurting a lot and I didn’t really feel like doing it at the time.” (ID6, teen)
	Reduce repetitive lesson content	“I tended to get a little frustrated because I want to get through the lesson and I wanted to learn, but it felt like I was learning the same things over and over again.” (ID2, parent) “I felt like it was repeating it too much, like if it was saying an example, then it would say, like, kind of the exact same examples in a couple more pages.” (ID9, teen)
	Enhance program accessibility and interactivity	“It would’ve been cool if it worked on a mobile phone or a mobile platform.” (ID1 parent) “One thing that could help is to kind of gamify it a little bit … maybe even sprinkled throughout add some multiple-choice questions and something just to kind of engage people.” (ID3 parent)

Five themes emerged highlighting the helpfulness and perceived benefit of the program’s delivery of several treatment components/skills, specifically: (1) cognitive and relaxation skills helped adolescents and parents cope with stress, (2) cognitive and relaxation skills helped adolescents cope with pain during recovery (3), strategies for improving sleep were beneficial both before surgery and during recovery, (4) activity pacing and goal-setting were used to gradually return to regular activities, and (5) parents valued strategies to encourage self-care. Two themes related to the perceived helpfulness of the general structure of the program were identified: (1) families found narratives relatable and validating and (2) families appreciated the flexibility of the online program. Though feedback was highly positive overall, participants provided suggestions for improvement with the most consistent feedback related to (1) rethinking timing and reducing the length of the first postoperative lesson, (2) reducing repetitive lesson content, and (3) enhancing program accessibility and interactivity.

#### Adverse Events

No adverse events were spontaneously reported during the study. As noted above, one adolescent participant needed to undergo major second surgery during the study period and thus the family was subsequently withdrawn from the study but allowed access to the intervention.

## Discussion

The goal of this pilot study was to examine the feasibility and acceptability of an Internet-based perioperative psychosocial treatment program to reduce acute and chronic postoperative pain among adolescents undergoing major surgery. Our findings confirmed feasibility across several metrics, including adequate recruitment and retention rates, high treatment engagement, and excellent assessment completion and retention. These data demonstrate our ability to effectively recruit patients and deliver an intervention program during both the preoperative and postoperative phases, a highly demanding time for families. Moreover, the intervention program was well received by adolescents and parents according to quantitative ratings and qualitative feedback. Families highlighted numerous strengths of the program as well as notable areas for improvement.

To our knowledge, this is the first pilot feasibility study of an Internet-delivered psychosocial intervention for adolescents undergoing major surgery. Despite the known risk for developing persistent postsurgical pain (CPSP^7,34^), very few perioperative psychosocial interventions have been evaluated in this population, and none have been delivered remotely via the Internet.^17^ There are programs delivered through smartphone applications to provide pain management strategies to adolescents focused on reducing acute postoperative pain,^35^ although program outcome data have not been published. Our intervention program is unique in targeting a broader range of known risk factors for the transition from acute to chronic pain, including adolescent and parent anxiety and sleep disturbance. The development of a flexible, accessible, and low-cost intervention delivered via remote technology to adolescents undergoing major surgery has the unique potential for widespread dissemination and broad reach, overcoming significant barriers related to access and family burden of in-person psychosocial interventions. Additional strengths of this pilot study include the development of both adolescent and parent versions of the intervention program, implementation during the preoperative and postoperative periods, and the use of quantitative and qualitative metrics to evaluate intervention acceptability.

Despite several strengths, this study also carries notable limitations. A single-arm pilot design was chosen to prioritize the evaluation of feasibility and acceptability, as is appropriate for research on newly developed interventions.^36^ However, this study design and the small sample size do not allow for an evaluation of treatment efficacy. The lack of a control group further limits the ability to determine the feasibility of randomization. We did not include a longer-term follow-up assessment and thus could not evaluate retention rate beyond 3 months. Moreover, five adolescent–parent dyads (42% of the sample) did not participate in the optional qualitative interview portion of the study. Finally, the majority of the sample comprised white females who underwent a single type of surgery at a single medical center in the northwestern United States. Although the sample demographics are representative of youth undergoing surgery,^37^ findings may not generalize to a more diverse population or those undergoing other types of major surgeries. Future studies will be needed to ensure that intervention engagement and acceptability remains high in more racially and socioeconomically diverse samples.

Given findings highlighting the feasibility and acceptability of this Internet-delivered psychological intervention program, an important next step is to evaluate the efficacy of the intervention in a full-scale randomized controlled trial. Indeed, given the favorable feasibility outcomes, we revised intervention components and developed a fully functional smartphone application and website to deliver the intervention in a multisite, fully powered randomized controlled trial (NCT04637802, ClinicalTrials.gov). For further details and trial protocol, please see Rabbitts et al.^38^ The larger trial uses a 2 × 2 factorial design to separately evaluate the two intervention phases (preoperative and postoperative) in order to expand understanding of the optimal timing of intervention delivery. Moreover, the trial uses an active comparator as a control: a psychoeducational program that provides information to families about preparation for and recovery from surgery but does not include direct training in cognitive-behavioral strategies. No major changes were made to the eligibility criteria or study intervention length; however, the final follow-up assessment was extended to 6 months to understand the durability of intervention effects in preventing the transition from acute to chronic pain.

Based on qualitative feedback from families, we made several modifications to enhance program features and delivery while maintaining the core intervention content. Most notable, the original program (previously delivered through REDCap) was transformed to be delivered via a mobile app for adolescents and a website for parents (called SurgeryPal), thus enhancing intervention accessibility and flexibility. We also reduced the length of the lessons and included several interactive features, including guided skill practice; symptom tracking, which triggers personalized “For You” content; and multimedia components (e.g., videos of parents and adolescents describing experiences and use of skills). Coaching calls were removed from the protocol to improve intervention scalability; instead, features unique to digital health platforms such as personalized notifications were included to encourage skill use and program completion. Moreover, based on participant feedback, we amended the delivery timing of the first postsurgical lesson from 1 week to 3 weeks after surgery to reduce participant burden during the immediate recovery period. For a more detailed description of the SurgeryPal app and website, see Rabbitts et al.^38^

There is an urgent need for effective and accessible psychosocial interventions for adolescents undergoing major surgery. The current study presents the first evaluation of a promising online psychosocial intervention delivered during the perioperative period to adolescents undergoing major surgery and their parents. Preliminary evidence demonstrates the high feasibility and acceptability of the intervention. The program’s content and online format were well received by families, and participant feedback was used to modify intervention elements and create an interactive digital health mobile app (for adolescents) and website (for parents) for the fully powered randomized controlled trial. Completion of the larger trial will be a crucial next step in understanding whether the SurgeryPal program effectively prevents CPSP and improves longer-term health outcomes among youth, with the potential for widespread integration into perioperative care.
